# Fructose-Drinking Water Induced Nonalcoholic Fatty Liver Disease and Ultrastructural Alteration of Hepatocyte Mitochondria in Male Wistar Rat

**DOI:** 10.1155/2015/895961

**Published:** 2015-07-26

**Authors:** Norshalizah Mamikutty, Zar Chi Thent, Farihah Haji Suhaimi

**Affiliations:** ^1^Discipline of Anatomy, Basic Medical Science Cluster, Faculty of Medicine, Universiti Teknologi MARA, Jalan Hospital, Sungai Buloh Campus, 47000 Sungai Buloh, Selangor, Malaysia; ^2^Department of Anatomy, Faculty of Medicine, Pusat Perubatan Universiti Kebangsaan Malaysia, Jalan Yaacob Latif, Bandar Tun Razak, 56000 Cheras, Kuala Lumpur, Malaysia

## Abstract

*Background.* Nonalcoholic fatty liver disease (NAFLD) is one of the complications of the metabolic syndrome. It encompasses a wide range of disease spectrum from simple steatosis to liver cirrhosis. Structural alteration of hepatic mitochondria might be involved in the pathogenesis of NAFLD.* Aims.* In the present study, we used a newly established model of fructose-induced metabolic syndrome in male Wistar rats in order to investigate the ultrastructural changes in hepatic mitochondria that occur with fructose consumption and their association with NAFLD pathogenesis.* Methods.* The concentration of fructose-drinking water (FDW) used in this study was 20%. Six male Wistar rats were supplemented with FDW 20% for eight weeks. Body composition and metabolic parameters were measured before and after 8 weeks of FDW 20%. Histomorphology of the liver was evaluated and ultrastructural changes of mitochondria were assessed with transmission electron micrograph.* Results.* After 8 weeks of fructose consumption, the animals developed several features of the metabolic syndrome. Moreover, fructose consumption led to the development of macrovesicular hepatic steatosis and mitochondrial ultrastructural changes, such as increase in mitochondrial size, disruption of the cristae, and reduction of matrix density.* Conclusion.* We conclude that in male Wistar rat 8-week consumption of FDW 20% leads to NAFLD likely via mitochondrial structural alteration.

## 1. Introduction

Obesity and fatty liver diseases are global health problems which involve not only adults but also children [1]. Usually, fatty liver disease is caused by consumption of alcohol. Hence, it is called alcoholic fatty liver disease (AFLD). However, causes not related to alcohol consumption which may cause fatty liver disease are obesity, diabetes mellitus type II, dyslipidemia, and hypertension, and it is known as nonalcoholic fatty liver disease (NAFLD) [1]. All these causes for NAFLD are also components of the metabolic syndrome. Hence, NAFLD is a manifestation of metabolic syndrome [1]. The risk of NAFLD is increased by 62% in an individual with newly diagnosed diabetes mellitus and 43% in an individual with impaired oral glucose tolerance test (OGTT) [2]. The most common cause of liver disease is NAFLD [2]. In western countries, approximately 20–30% of the populations suffer from NAFLD. Although NAFLD is a benign condition, it may contribute to morbidity and mortality [2].

Alteration in mitochondria also known as “cellular power plants” plays an important role in the pathogenesis of NAFLD. It is an organelle involved in oxidation of lipid and energy production from fat and glucose into adenosine triphosphate (ATP) to be used by the cells. The hepatocytes' cytoplasm contains numerous mitochondria, which represent approximately 18% of liver volume [3].

Dietary habits of the populations worldwide have changed [4]. There has been an increase in consumption of soft drinks which has led to an increase in fructose consumption and an increase in energy intake from 3.9% to 9.2% in 2001 [5]. Various studies suggest that fructose consumption is associated with the development of metabolic syndrome [6–9]. Fructose stimulates hepatic lipogenesis and synthesis of triglyceride [10]. Thus, consumption of fructose for a long period leads to formation of macrovesicular and microvesicular steatosis [11]. In animal models, consumption of high carbohydrate and high fat diet for 16 weeks leads to the development of metabolic syndrome and hepatic steatosis [12]. A recent study has shown that in male Wistar rats consumption of drinking water with 20% of fructose for 8 weeks resulted in an increase in body weight and body mass index and in the development of several features of metabolic syndrome such as central obesity, dyslipidemia, hypertension, and hyperglycemia [13]. This model of metabolic syndrome induced by dietary modification is similar to what happens in humans. Thus, in the present study, we used a newly established model of fructose induced metabolic syndrome to investigate the changes in morphology of the hepatic mitochondria that occur with fructose consumption and their role in NAFLD pathogenesis. We hope that a better understanding of the pathogenesis of NAFLD induced by fructose will help further investigations in this field.

## 2. Materials and Methods

### 2.1. Physiological Measurements

The percentage of body weight increment, body mass index (BMI), abdominal circumference, blood pressure, fasting plasma glucose, fasting lipid profile, and abdominal adipose tissue were measured at baseline and at the end of experiment study. The experiment protocol was in accordance with an earlier study [13].

### 2.2. Animals and Diets

Twelve male Wistar rats (250–300 grams) were obtained from the Animal House of Universiti Kebangsaan Malaysia and used in this study. The animals were housed in individual cages under control conditions of temperature (20–22°C) and lighting (12 light/12 h dark). The rats were randomly divided into two groups, the control group (C) and fructose-drinking water group (F20). There were six rats in each experimental group. Both groups were fed with normal rat chow diet; however, they differ in water intake. Control group (C) was administered tap water while the F20 group was administered 20% fructose in the drinking water. The food and water intake was given* ad libitum* for eight weeks. All experimental procedures were performed according to the regulations of the Animal Ethics Committee of Universiti Kebangsaan Malaysia (FP/ANAT/2012/FARIHAH/18-JULY/453-JULY-2012-AUGUST-2013).

### 2.3. Preparation of Fructose-Drinking Water

The preparation of 20% fructose in the drinking water was similar to a past study [13]. The fructose used was D-fructose >99% (Syarikat System Malaysia).

### 2.4. Liver Enzymes

Blood samples were attained at baseline and at end of the experiment via orbital vein in anaesthetized rats. The samples of blood were put in a plain bottle. Then, centrifugation was done at 3000 r.p.m. for 10 minutes at 4°C. The plasma samples were separated in the Eppendorf tube and were sent to the Pathlab for blood biochemistry analysis for aspartate transferase (AST) and alanine aminotransferase (ALT).

### 2.5. Liver Weight

The rats were sacrificed by inhalation of diethyl ether [14]. A longitudinal incision was done at the ventral aspect of the body. The liver was removed and dried with gauze before weighing. The weight of liver was normalised to tibial length and expressed as milligram per millimetre tibial length (mg/mm) [15].

### 2.6. Histology of Liver

#### 2.6.1. Hematoxylin and Eosin

The liver tissues were immediately fixed in 10% formalin for three days. These tissue samples were processed using an automatic-tissue processing machine and followed by embedding in paraffin wax. Thin sections (5 *μ*m) were obtained and stained with hematoxylin and eosin (H&E). Finally, the sections were mounted on dibutyl phthalate in xylene (DPX). The slides were viewed under light microscope at magnification ×20. Photomicrographs of the liver sections were captured.

#### 2.6.2. Oil Red O

The ORO staining was done by using paraffin embedding samples [16]. The liver tissues were fixed in 10% formalin for three days. These tissue samples were processed using an automatic-tissue processing machine and followed by embedding in paraffin wax. Thin sections (5 *μ*m) were obtained and mounted on a glass slide. To detect neutral lipid accumulation, sections were stained with Oil Red O for 15 minutes, counterstained with Mayer's hemalum for 5 dips, and coverslipped with a DPX. The slides were viewed under light microscope at magnification ×20. Photomicrographs of liver were taken.

#### 2.6.3. Transmission Electron Microscope

The liver tissues were immediately dissected into 1 mm³ cubes and placed into 3% glutaraldehyde for 5 minutes at 4°C. The samples then were washed with PBS buffer 0.1 M for three times, postfixed 2 hours at room temperature in 1% osmium tetroxide, blocked for 1 hour in 3% uranyl acetate, dehydrated through graded series of ethanol and acetone, infiltrated with 100% resin for 12 hours, and embedded in the 100% resin beam capsule. Then, the samples were polymerised in the 60°C oven for 12 hours before semithin sections were done at 500 nm. Following that, the samples were stained with toluidine blue for 20 seconds and were examined under light microscope to choose the area needed for further ultrathin sectioning at 70–90 *μ*m. The ultrathin sections were then put in the grid and then stained with uranyl acetate for 10 minutes and plumbum citrate for 5 minutes. Finally, the samples were viewed under electron microscope for lipid inclusion in the hepatocytes cytoplasm and morphology of mitochondria.

### 2.7. Statistical Analysis

All data were analysed using Statistical Package Social Sciences (SPSS) version 20 software and were presented in mean ± SEM subjected to one-way ANOVA. Value of *P* < 0.05 was taken as significant.

## 3. Results

Table 1 shows the physiological changes in both groups. After consumption of FDW 20% for eight weeks, the rats showed significant differences in metabolic profile which fulfil the criteria of metabolic syndrome. The rats in the F20 group became obese as evidenced by an increase in body weight, in BMI, in abdominal circumference, and in deposition of abdominal adipose tissue. These rats also developed hypertension, hyperglycemia, and hypertriglyceridemia.

**Figure 1:**
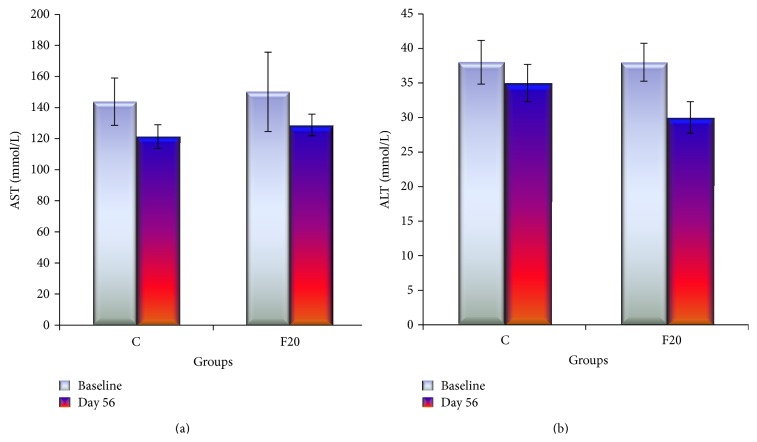
The bar graph of liver enzymes AST and ALT showed that there was no significant difference of the liver enzymes in between C and F20 at day 56 and there was no significant difference within each group at baseline and day 56.

**Figure 2:**
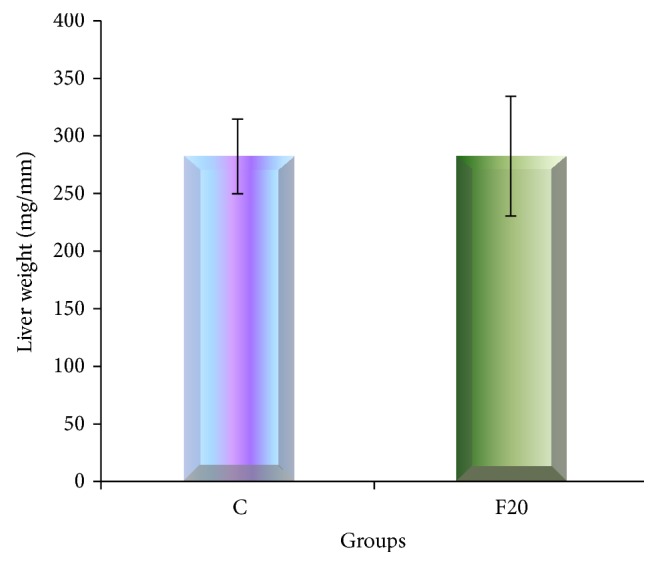
The bar graph of liver weight showed that there was no significant difference of the weight of the liver in between C and F20.

The AST enzyme was slightly higher in F20 compared to C group following consumption of FDW 20% for eight weeks but there was no statistically significant difference. The ALT enzyme was not increased at the end of the experiment in F20 group (Figure 1). Consumption of FDW 20% for eight weeks resulted in minimal increment of liver weight, which was not significantly different from the C group (Figure 2).

**Figure 3:**
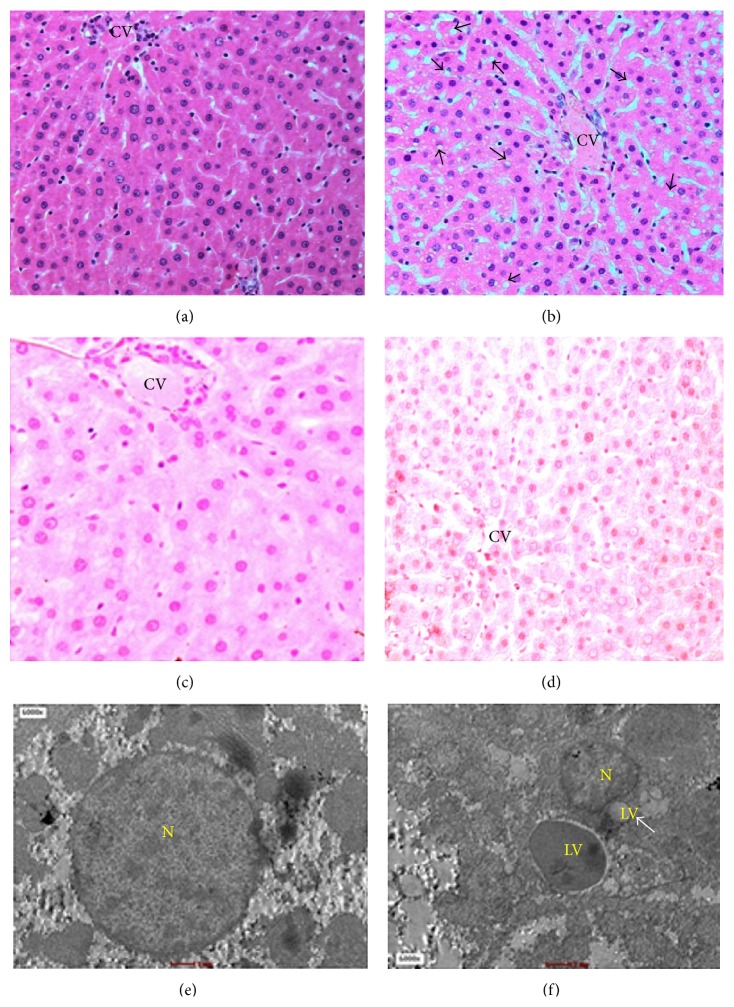
Hematoxylin and eosin staining of liver showed depositions of lipid vacuoles (*arrow*) in hepatocyte cytoplasm of F20 (b). Special staining of lipid vacuoles by ORO stained the lipid vacuoles with red and the nucleus of hepatocyte with blue in the F20 (d). TEM of F20 (f) showed the distorted nucleus of hepatocyte and multiple lipid depositions in the cytoplasm. No deposition of lipid vacuoles seen in the C group. N-nucleus and LV-lipid vacuoles.

Figure 3 shows hepatic steatosis with evidence of deposition of lipid vacuoles in the cytoplasm of hepatocyte after consumption of FDW 20% for eight weeks. The lipid vacuoles were macrovesicular type as characterized by the presence of single lipid vacuole. Majority of lipid vacuoles were arranged either in singlet or in groups of 2-3 vacuoles. The nucleus of the lipid vacuoles were pushed to the periphery; hence, it gives an appearance of a signet ring. The depositions of the lipid vacuoles were greatest in zone III followed by zone II and least in zone I. There was neither ballooning degeneration nor inflammatory cells seen.

**Figure 4:**
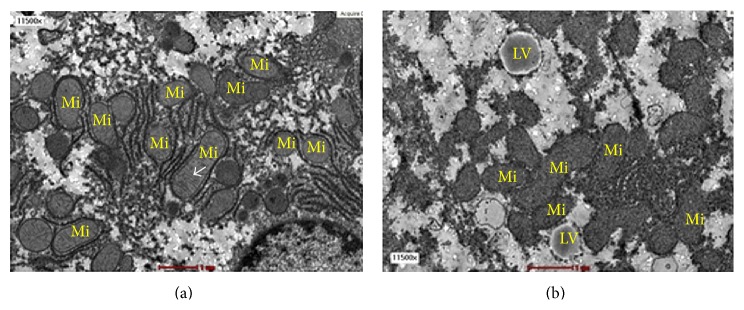
The transmission electron micrograph of hepatocytes from C group (a) and F20 group (b) at magnification ×11 500. The F20 group developed hepatic steatosis. The mitochondria from F20 group were bigger in sizes with swollen, disrupted cristae and hypodense matrix. Mi-mitochondria and LV-lipid vacuole. The* arrow* shows the cristae.

The mitochondria in F20 group showed ultrastructural abnormalities associated with hepatic steatosis (Figure 4). In the C group, the size of mitochondria ranged between 0.5 and 2 *μ*m. The cristae of the mitochondria were well defined and arranged closely to one another. Mitochondrial matrix of the C-group was denser compared to F20 group. In contrast, in the F20 group, the mitochondria showed increase in size which was between 2.5 and 3 *μ*m. The mitochondria cristae were disrupted and sparse between one another and the matrix was decreased in density or hypodense.

## 4. Discussion

Several criteria of the metabolic syndrome, such as central obesity, hyperlipidemia, hypertension, and hyperglycemia, developed after consumption of FDW 20%. These results are in accordance with a previous study [13]. Following eight weeks of consumption of FDW 20%, depositions of lipid vacuoles in the hepatocyte cytoplasm occurred. Nevertheless, the weight of the liver was not increased. The ways of distribution of lipid vacuoles were responsible for explaining the reason that contributed to the weight of the liver. Present study showed that the lipid vacuoles were distributed in hepatocytes cytoplasm either singular or doublet. Furthermore, there were no inflammatory cells seen. Thus, there were no changes in the liver weight as the weights of the lipid vacuoles were evenly distributed throughout the liver. In comparison with earlier research, the liver weight was increased and there was presence of inflammatory cells as well as the development of fibrosis after consumption of high-fat, high-carbohydrate (HFHC) diet for eight weeks in male Wistar rat [12]. We hypothesized that the increase in the liver weight could be attributed to the higher numbers of lipid vacuoles depositions in the hepatocyte cytoplasm and the presence of fibrosis that may have changed the consistency of the liver following consumption of HFHC diet compared to FDW 20% only.

The divisions of liver zones are according to the liver acinus classification because it relates to the liver structure with the blood perfusion, metabolic activity, and pathology of liver [3]. The lipid vacuoles were deposited mainly in zone III followed by zone II and the least in zone I. Zone III showed the highest lipid depositions because it was the farthest zone from the portal vein. Thus, it is the last zone to receive blood perfusion and nutrients from the portal vein. Liver steatosis in obesity manifests as macrovesicular type and has signet ring appearance [1]. The results of the present study are in accordance with an earlier study which showed that the lipid inclusions were of macrovesicular type where the nucleus was pushed to the periphery and gave the characteristic of signet ring cell appearance. In the microvesicular type, there are multiple lipid vacuoles and the nucleus are centrally located [17].

There are three enzymes produced by the hepatocytes, namely, ALT, AST, and alkaline phosphates (ALP). In the inflammation state of the liver, both the ALT and AST enzymes are raised. However, the AST enzyme is not specific for the liver as it is also been produced by the cardiac cells. Thus, the present study did not measure the ALP enzyme. This is due to the facts that ALP enzyme elevated in an obstruction of biliary ducts such as in intrahepatic cholestasis. The liver enzymes (AST and ALT) of the experiment were measured at the end of the experiment. There was no elevation of liver enzymes and this was consistent with the histological finding that showed no inflammatory cells. In contrast to a previous study, there was an increase in liver enzymes which was consistent with the presence of inflammatory cells and fibrosis in the liver [12]. We hypothesized that consumption of FDW 20% more than eight weeks may lead to the inflammation of liver cells and elevation of liver enzymes.

The changes in the structure of mitochondria in NAFLD by using TEM also were observed following consumption of FDW 20. Mitochondria are organelles involved in lipid oxidation and production of ATP from fat and glucose to be used as energy by cells. Each hepatocyte contains 800 or 18% of the total cell volume. Mitochondria play an important role in the hepatocyte metabolism and act as a primary site for fatty acid oxidation. The mitochondria possess an oval or elliptical shape with size ranging between 0.5 and 2 *μ*m [18]. There are two phospholipids and protein membranes covering the mitochondria, namely, inner and outer membranes [3]. There are several enzymes present in the matrix of mitochondria which contribute to the density of the matrix. In TEM, the matrix of normal mitochondria is identified as hyperdense area [17]. One of the major functions of the enzyme in the matrix is meant for fatty acid oxidation. The mtDNA that is present in the matrix is very sensitive to the oxidative stress. Thus, any factor that interferes with the structure of the mitochondria may lead to disturbances in its function [17].

Mitochondrial dysfunction might contribute to the pathogenesis of NAFLD because of disturbances in mtDNA and in lipid oxidation function [17]. The changes in the morphology of the mitochondria include increase in its size, loss of the cristae, and hypodensity of the matrix [17]. In the present study, the mitochondria in F20 group showed morphological disruption whereby the sizes were increased, the cristae were disrupted, and the matrixes were hypodense compared to C group. All these changes were consistent with the mitochondria dysfunction which was due to accumulation of lipid vacuoles in the hepatocyte cytoplasm.

Morphologically, there are two steps involved in the formation of megamitochondria [19]. The first step involves simple swelling of the mitochondria which can be followed by the formation of megamitochondria. In simple swelling, the size of mitochondria does not exceed three times compared to the control group. However, in megamitochondria, the size of the mitochondria is three times larger as compared to control. This is due to a tear of the outer membrane of the mitochondria which leads to fusion with other mitochondria around it. The present study demonstrates that consumption of FDW 20% for eight weeks resulted in simple swelling of mitochondria rather than megamitochondria. Perhaps, if the duration of consumption of FDW is prolonged, it may lead to formation of megamitochondria.

There are several stages of NAFLD natural history, ranging from simple steatosis to steatohepatitis (NASH), liver cirrhosis, and finally carcinoma of the liver [20]. The first stage, which is steatosis, is characterized by the presence of lipid inclusion in the liver. In NASH, steatosis is accompanied by inflammatory cells, ballooning of the hepatocytes, and often elevation of liver enzymes. Fibrosis is present when cirrhosis develops and is due to liver cells death. The present study shows that eight-week consumption of FDW 20% resulted in the development of simple steatosis, the first stage of NAFLD. This was evidenced by the presence of lipid inclusions without inflammatory cells and normal liver enzymes. Previous studies have shown that consumption of HCHF diet resulted in more advanced stage of NAFLD [12].

## 5. Conclusion

We conclude that in male Wistar rats eight weeks of high fructose consumption caused the development of the metabolic syndrome and the formation of NAFLD, via morphological changes in hepatic mitochondria. One important limitation of our study is the lack of assessment of molecular mitochondrial function, which needs to be addressed in future studies, to better understand the association between alterations in mitochondrial morphology and function. Moreover, future studies are needed to investigate the effects of doses of fructose consumption more relevant to people.

## Figures and Tables

**Table 1:** Effects of fructose-drinking water on metabolic variables in C and F20 groups for 8 weeks.

Variables	C		F20	
	Baseline	8 weeks	Baseline	8 weeks
Body weight gain (%)	—	36.12 ± 0.81	—	43.03 ± 0.76^a^
Body mass index (g/cm^2^)	0.64 ± 0.01	0.66 ± 0.02	0.64 ± 0.02	0.91 ± 0.02^ab^
Abdominal circumference (cm)	16.5 ± 0.26	18.3 ± 0.1	16.1 ± 0.40	22.9 ± 0.3^ab^
Total abdominal fat (mg/mm tibial length)	—	196.72 ± 23.13	—	437.97 ± 27.08^a^
Plasma triglyceride (mmol/L)	0.70 ± 0.09	0.65 ± 0.08	0.78 ± 0.07	1.22 ± 0.14^ab^
Plasma total cholesterol (mmol/L)	1.5 ± 0.01	1.6 ± 0.1	1.5 ± 0.04	1.5 ± 0.1
Systolic blood pressure (mmHg)	103.3 ± 1.1	105.0 ± 1.8	101.3 ± 2.1	145.8 ± 1.5^ab^
Plasma glucose (mmol/L)	5.1 ± 0.4	6.4 ± 0.2	4.7 ± 0.4	8.1 ± 0.6^ab^

Values are mean ± SEM and *n* = 6 for each group. Superscripts letters are significantly different. ^a^A significant difference as compared to C group at 8 weeks. ^b^A significant difference within groups as compared to baseline.
